# Stimulant Use Associated With Psychosocial Factors, HIV Risk, and Concurrent Hazardous Alcohol Use Among US Adults: Exploratory Cross-Sectional Questionnaire Study

**DOI:** 10.2196/45717

**Published:** 2023-08-17

**Authors:** Frank Lee, Jennifer Payaal Jain, Lunthita M Duthely, Janet Ikeda, Glenn-Milo Santos

**Affiliations:** 1 Center on Substance Use and Health San Francisco Department of Public Health San Francisco, CA United States; 2 Department of Psychiatry and Behavioral Sciences School of Medicine University of California San Francisco San Francisco, CA United States; 3 Obstetrics, Gynecology & Reproductive Sciences Miller School of Medicine University of Miami Miami, FL United States; 4 Department of Public Health Sciences University of Miami Miami, FL United States; 5 Department of Community Health Systems School of Nursing University of California San Francisco San Francisco, CA United States; 6 Division of Prevention Science School of Medicine University of California San Francisco San Francisco, CA United States

**Keywords:** Amazon Mechanical Turk, stimulant use, alcohol use, craving, men who have sex with men, MSM, depression, affect, HIV, public health, gender minority, psychosocial, addiction

## Abstract

**Background:**

Stimulant use is a major public health problem that contributes to morbidity and mortality among men who have sex with men (MSM) in the United States. To reduce the harms associated with stimulant use, there is a need to identify the factors associated with stimulant use to inform interventions. Additionally, there is a need to use large crowdsourcing platforms like Amazon Mechanical Turk (MTurk) to engage more individuals who use substances across the United States.

**Objective:**

We identified the correlates of stimulant use among people who use alcohol or stimulants in the United States recruited using MTurk.

**Methods:**

Participants who were aged ≥18 years in the United States and reported alcohol or stimulant (ie, cocaine, crack cocaine, and methamphetamine) use were deemed eligible and recruited via the web platform MTurk. Participants completed a baseline survey, which assessed sociodemographics, psychosocial (ie, depression, affect, self-esteem, and stress) factors, substance use, and sexual behaviors. Data were collected and analyzed with STATA (version 17; StataCorp). Stratifying by MSM status, bivariate and multivariable logistic regression models were built in STATA to examine the correlates of stimulant use. Multivariable models controlled for age, race, health insurance, and relationship status.

**Results:**

Of 272 participants, 201 (73.9%) identified as male, 134 (49.2%) were MSM, 52 (19.1%) were from racial and ethnic minoritized communities, and 158 (58%) were in a relationship. The mean age was 36.10 (SD 10.3) years. A total of 40 (14.7%) participants reported stimulant use in the past 6 months. Factors significantly associated with stimulant use were being MSM (adjusted odds ratio [aOR] 4.61, 95% CI 1.97-10.81), a higher Alcohol Use Disorders Identification Test-Concise score (aOR 1.24, 95% CI 1.08-1.42), more intense cravings for alcohol in the past 24 hours (aOR 1.03, 95% CI 1.01-1.04), a higher depression score (aOR 1.06, 95% CI 1.01-1.12), a greater number of male partners in the last 6 months (aOR 1.32, 95% CI 1.08-1.61), a greater number of female partners in the last 6 months (aOR 1.42, 95% CI 1.04-1.92), and being diagnosed with a sexually transmitted infection (eg, syphilis, gonorrhea, chlamydia, herpes simplex virus, human papillomavirus, and other) in the last 6 months (aOR 14.61, 95% CI 3.45-61.87). Additionally, there was a significant additive interaction between MSM status and negative affect, such that the impact of negative affect on stimulant use was significantly greater among MSM compared with non-MSM (relative excess risk due to interaction 0.085, 95% CI 0.037-0.13).

**Conclusions:**

Interventions that address stimulant use should use evidence-based approaches that reduce negative affect, depression, and cravings for alcohol. Additionally, interventions should be customized for MSM populations.

## Introduction

Stimulant use, defined as the use of methamphetamines, cocaine, or crack cocaine, is a major public health problem and a leading cause of morbidity and mortality in the United States [1,2]. In the general population, stimulant use has been observed among multiple groups including college students [3], persons who inject drugs [4], and unhoused individuals [5]. Importantly, stimulant use is more prevalent among men who have sex with men (MSM) when compared with the general population [6-8]. Stimulant use is associated with HIV risk behaviors (eg, condomless sex and multiple sex partners) and HIV incidence among MSM [9,10]. The use of stimulants has also been increasing among individuals with opioid use disorders and been associated with increased risk of drug overdoses [11].

Among MSM, stigma, discrimination, and experiences of prejudice have been linked to stress and poor mental health conditions including low self-esteem, negative affect, and depression [12]. Furthermore, stress and poor mental health have been linked to higher rates of substance use among MSM [13]. Substance use has been identified as a form of self-medication among MSM to deal with stress and trauma associated with being a sexual minority in the United States [14,15]. The minority stress theory, which posits that MSM experience substantial stress due to societal discrimination, stigma, and prejudice toward their sexual orientation, is particularly relevant in this context and informs this research [16].

People who use substances are often difficult to be recruited and retained in research. Hence, there is a need to leverage novel data collection strategies that can maintain participant confidentiality and privacy, especially given the criminalization and stigmatized nature of substance use. Amazon Mechanical Turk (MTurk), an innovative crowdsourcing platform, addresses these issues by recruiting large populations who use substances over the web platforms [17]. MTurk, which remains underused in substance use and HIV research, could be used more often to engage larger populations in the United States that can be underrepresented using traditional survey methods [17]. Despite the unique stressors and trauma that affect MSM and their substance use, studies that have examined differences among MSM and non-MSM remain limited. Taken together, there is a need to leverage crowdsourcing platforms like MTurk to recruit MSM who use stimulants into research to identify the demographic, behavioral, and psychosocial predictors of stimulant use among this population to inform substance use prevention and treatment efforts [18-20].

To address this gap in research, we used the minority stress theory to guide our work and identified the correlates of stimulant use among MSM and non-MSM in the United States recruited via MTurk. Consistent with the minority stress theory, we posited that negative affect, depression, low self-esteem, and high stress would be associated with stimulant use and that the impact of these factors on stimulant use would be stronger among MSM compared with non-MSM. Understanding the demographic, behavioral, and psychosocial factors that are associated with stimulant use may help inform the development of interventions that reduce stimulant use and related harms among MSM.

## Methods

### Ethics Approval

The Stimulant and Alcohol Use in MTurk Behavioral Assessments Study (SAMBA) is a study looking at substance use and HIV risk factors among both MSM and non-MSM in the United States; our study uses baseline data from the SAMBA study. During the screening process, informed consent was provided via a web-based information sheet that contained information about the purpose, study procedures, risks and benefits from participation, and protection of confidentiality. The institutional review board at the University of California, San Francisco reviewed and approved the SAMBA study protocol (IRB study number 16-20295).

### Recruitment, Screening, and Enrollment

A total of 272 participants were recruited digitally from an existing pool of active MTurk workers between March 26, 2018, and April 13, 2018. As the parent study was an exploratory study assessing the prevalence of various behavior and risk factors, participation in the study was limited to a maximum of 300 participants (150 non-MSM and 150 MSM). After responding to the initial screening survey posted as a Human Intelligence Task on MTurk, participants were screened into the study. Participants were eligible if they (1) were at least 18 years old, (2) able to speak English, and (3) reported alcohol or stimulant (ie, cocaine, crack cocaine, or methamphetamine) use in the past year. Two eligible groups were recruited by the SAMBA study: MSM who use alcohol or stimulants and non-MSM who use alcohol or stimulants. Because the MSM population is disproportionately impacted by both substance use and HIV-related risk behaviors, the parent study sampled MSM explicitly [7,8].

### Web-Based Surveys

Web-based surveys were completed by the participants via computers or smartphones. To screen for eligibility, an initial survey with a compensation of US $0.80 was completed. If eligible and agreed to informed consent, a baseline survey with a compensation of US $5 was completed. Individual MTurk accounts were set up for each participant, and research staff provided links through these accounts to complete surveys using a unique authenticator (single sign-on token).

### Measures

#### Sociodemographics

Sociodemographic factors were assessed, including age in years, race (White, Asian, African American or Black, Native American or Alaskan Native, Hawaiian or Pacific Islander, and other), and ethnicity (non-Hispanic or Latino and Hispanic or Latino). To assess being MSM, a dichotomous measure was created from the following two questions: (1) “What was your sex at birth (male or female)?” and (2) “Who do you have sex with (men, women, transgender females or transwomen, or transgender males or transmen)?” Additionally, other factors such as relationship status (married or committed, single, and divorced), employment status (full-time, part-time, and unemployed), having health insurance (yes or no), ever tested for HIV (yes or no), and annual income (≥US $125,000, US $75,000-124,999, US $40,000-74,999, and ≤US $40,000) were assessed. Finally, a dichotomous measure of higher education was created by distinguishing between those having at least a 4-year degree (attained a bachelor’s degree or completed any postgraduate studies) versus those who completed 12th grade or general education degree, or an associate of arts degree or some college.

#### Alcohol Use

To address alcohol use, alcohol consumption in the past 6 months (yes or no) and current or past hazardous drinking (yes or no) were identified using the 3-item Alcohol Use Disorders Identification Test-Concise (AUDIT-C); scores ≥4 for male individuals and ≥3 for female individuals indicate hazardous drinking [21,22]. The AUDIT-C is a 3-question scale used to identify problematic alcohol use, with scores ranging from 0 to 12 (a score of 0 reflects no alcohol use in the past year) [21,22]. Having a higher AUDIT-C score represents an increased likelihood that one’s drinking is harming their health [21,22].

#### Substance Use

To assess substance use, questions about methamphetamine use, cocaine use, crack cocaine use, and any drug use, which included reporting any injection or noninjection drug use in the past 6 months (yes or no), were also asked. For this analysis, our primary outcome was stimulant use in the past 6 months, which is defined as reporting at least one of the following: methamphetamine use in the past 6 months, powdered cocaine in the past 6 months, or crack cocaine in the past 6 months. These measures are consistent with the windows for different substance use classes used by the National Survey on Drug Use and Health, which has demonstrated high reliability and substantial agreement in test-retest evaluations measuring up to 12 months of recall [23].

#### Psychosocial Measures

To assess psychosocial measures in our sample, we used various psychosocial validated scales that have been used in other MSM-focused studies. To assess self-esteem, the 10-item Rosenberg Self-Esteem Scale (RSES) was used. The RSES scale has been used previously to look at HIV risk factors among MSM populations [24]. To assess positive and negative affect, the 20-item Positive and Negative Affect Schedule (PANAS) scale was used. The PANAS scale has been used in a study looking at the association between affect and sexual behavior among MSM individuals [25]. To assess perceived stress, the 10-item Perceived Stress Scale (PSS) was used. The PSS scale has been validated in a previous study that assessed the legitimacy of the PSS scale as a measure of stress among lesbian, gay, bisexual, transgender, queer/questioning, and other (LGBTQ+) individuals [26]. To assess depression, the 10-item Center for Epidemiologic Studies Depression Scale (CESD-10) scale was used. The CESD-10 scale has been used in a study assessing depression and suicidality among MSM and transgender women [27].

- Self-esteem: Using the RSES scale [28,29], self-esteem was assessed, where greater scores represent greater levels of self-esteem. General feelings of self-esteem were assessed with levels of agreement with statements about self-esteem (α=.92). A 4-point scale ranging from “strongly agree” to “strongly disagree” was used, and items 2, 5, 6, 8, and 9 were reverse coded to make sure that higher scores mapped to higher levels of self-esteem. Final scores were assessed via the summation of all 10 items.
- Positive and negative affect: Using the PANAS scale [30], positive affect and negative affect were measured, with scores ranging from 10 to 50 and greater scores indicating greater levels of positive (α=.90) or negative (α=.94) affect. Participants recorded their level of agreement with the 20 emotions assessed on the PANAS via a 5-point scale (1=very slightly or not at all to 5=extremely). Negative affect was calculated by summing items 2, 4, 6, 7, 8, 11, 13, 15, 18, and 20. Positive affect was calculated by summing scores from 1, 3, 5, 9, 10, 12, 14, 16, 17, and 19.
- Perceived stress: Using the PSS scale [31], stress was measured, with scores ranging from 0 to 40 and greater scores representing greater levels of perceived stress. Participant experiences and frequencies of different feelings and thoughts were assessed via a 5-point scale (0=never to 4=very often), with items 4, 5, 7, and 8 reverse coded to ensure that greater scores mapped onto greater levels of stress. By summing all items, a total stress score was calculated. Finally, 3 categories of low stress (scores range from 0 to 13), moderate stress (scores range from 14 to 26), and high stress (scores range from 27 to 40) were also measured using the PSS.
- Depression: Using the CESD-10 scale [32], depression was measured, with scores ranging from 0 to 30. Per the CESD-10, scores ≥10 are considered to represent depression (α=.91). Participants reported the frequency of various emotional states using a 4-point scale (0=rarely, or none of the time or less than 1 day to 3=all of the time or 5-7 days) in the last week. In error, item 10 (“I could not get going”) was not included in the questionnaire, so scoring for CESD-10 was performed by averaging items 1 to 9. To ensure that higher scores mapped onto higher levels of depression, items 5 and 8 were reverse coded. A total depression score was calculated by summing all 9 items.

#### Cravings for Alcohol and Stimulants

Participants were asked to report cravings for alcohol and stimulants (ie, cocaine, crack cocaine, and methamphetamine) in the past 24 hours if applicable. Scores ranged from 0 to 100, with 0 representing no craving whatsoever and 100 representing the strongest craving one has ever had.

### Statistical Analysis

#### Descriptive Statistics

Data were analyzed using STATA (version 17; StataCorp). Using descriptive statistics including frequencies, percentages, and depending on the distributional assumption, means, SDs, medians, and IQRs, we described the study sample overall and by subgroup (MSM vs non-MSM). Then, using χ^2^ tests for categorical variables, *t* tests for continuous variables, and Wilcoxon signed rank sum tests for not normally distributed continuous variables, we identified which variables varied significantly across the subgroups (Table S1 in Multimedia Appendix 1).

#### Bivariate Logistic Regression

Using bivariate logistic regression, we examined which factors in Table S1 in Multimedia Appendix 1 were associated with stimulant use among each subgroup separately by building stratified models (Table S2 in Multimedia Appendix 2).

#### Multivariable Logistic Regression

We then built multivariable logistic regression models to identify the correlates of stimulant use after controlling for age, race, health insurance, and relationship status, known correlates of substance use in the scientific literature [33-36]. Importantly, we modeled each primary exposure separately and built separate models for MSM and non-MSM (Table S3 in Multimedia Appendix 3).

#### Interaction Analyses

Finally, to test MSM status as a moderator of the relationship between negative affect and stimulant use, we estimated the relative excess risk due to interaction (RERI) using an additive scale while controlling for confounders [37].

## Results

### Baseline Characteristics

The baseline characteristics of this study are described in Table S1 in Multimedia Appendix 1. Of 272 recruited participants, 134 (49.2%) identified as MSM, and the mean age was 36.1 (SD 10.3) years. A total of 52 (19.2%) participants were people of color, with the following breakdown: African American or Black (22/52, 42%), followed by Asian (17/52, 33%), other (10/52, 19%), and then Native American or Alaskan Native (3/52, 6%). In total, 248 (91.1%) identified as non-Hispanic or Latino, and 24 (8.8%) identified as Hispanic or Latino. A total of 201 (73.9%) were male and 71 (26.1%) were female. A little more than half were married or in a committed relationship (158/272, 58%), whereas less than half reported being single (112/272, 41.1%), and only 2 (0.7%) participants were divorced or separated. Most participants (180/272, 66.1%) were fully employed, and over half (163/272, 59.9%) reported having attained higher education defined as completion of at least a 4-year program or any other postgraduate studies. Income levels were split, with slightly over one-third (n=99, 36.4%) earning less than US $40,000, 89 (32.7%) earning US $40,000-$74,999, 64 (23.5%) earning US $75,000-$124,999, and only 20 (7.3%) earning US $125,000 or more. The majority (229/272, 84.1%) of the sample reported having health insurance, and over half (170/272, 62.5%) reported ever being tested for HIV. Some (95/272, 34.9%) reported never having been tested for HIV, and a few (7/272, 2.5%) were not sure or did not know. Lastly, geographically, participants were from the Northeast (46/272, 17%), Southeast (95/272, 35%), Midwest (65/272, 24%), and Southwest or West (65/272, 24%).

Nearly all participants (261/272, 95.9%) reported consuming alcohol within the past 6 months. The mean AUDIT-C score was 4.2 (SD 2.4), and 173 (63.6%) participants met the criteria for hazardous alcohol consumption based on AUDIT-C scores. In the past 6 months, 19 (6.9%) participants reported using methamphetamine, 31 (11.3%) reported using powdered cocaine, 8 (2.9%) reported using crack cocaine, and 104 (38.2%) reported any noninjection or injection drug use. A total of 40 individuals (14.7%) reported stimulant use in the past 6 months.

On a scale from 0 to 100, median day-level cravings for alcohol, methamphetamine, cocaine, and crack cocaine were 5 (IQR 0-26), 54 (IQR 20-88), 39 (IQR 1-71), and 52 (IQR 51-87), respectively. On a scale from 10 to 40, the median score for self-esteem was 30 (IQR 26-35). On a scale from 10 to 50, median scores for positive affect and negative affect were 31 (IQR 26-37) and 15 (IQR 11-20), respectively.

On a scale from 0 to 40, the median perceived stress score was 17 (IQR 11-21), and a little over half (152/272, 55.8%) of the sample reported experiencing moderate stress, followed by low stress (93/272, 34.1%) and high stress (27/272, 9.9%). On a scale from 0 to 30, where a score ≥10 is considered to indicate depression, the median score was 7.7 (IQR 3.3-12.2). A total of 115 (42.2%) participants had depressive symptoms, with a CESD-10 score greater than or equal to 10.

### Sociodemographic Differences by MSM Status

There were some notable sociodemographic differences between MSM and non-MSM participants. MSM participants were significantly younger than non-MSM (mean 34.23, SD 10.5 years vs mean 37.92, SD 9.8 years; *P*=.003), more likely to be single (73/272, 26.8% vs 39/272, 14.3%; *P*<.001), and more likely to identify as Hispanic or Latino (18/272, 6.6% vs 6/272, 2.2%; *P*<.008). MSM participants were also less likely to be in higher income brackets than non-MSM (US $75,000-$124,999, 21 vs 43, and ≥US $125,000, 8 vs 12, respectively; *P*=.01; Table S1 in Multimedia Appendix 1).

### Substance Use and Cravings Differences by MSM Status

Compared with non-MSM participants, MSM participants had a greater mean AUDIT-C score (mean 4.7, SD 2.6 vs mean 3.7, SD 2.1; *P*<.001) and were more likely to report using methamphetamine (16/272, 5.8% vs 3/272, 1.1%; *P*<.001), cocaine (25/272, 9.1% vs 6/272, 2.2%; *P*<.001), crack cocaine (6/272, 2.2% vs 2/272, 0.7%; *P*=.50), stimulants (32/272, 11.7% vs 8/272, 2.9%; *P*<.001), and any noninjection or injection drug use (68/272, 25.0% vs 36/272,13.2%; *P*<.001) in the past 6 months. Additionally, MSM participants had higher median day-level craving for alcohol (median 8, IQR 0-30 vs median 2, IQR 0-23; *P*=.048) in the past 24 hours (Table S1 in Multimedia Appendix 1).

### Psychosocial Factor Differences by MSM Status

Compared with non-MSM participants, MSM participants had lower median self-esteem scores (median 29, IQR 24-33 vs median 30, IQR 28-36; *P*=.008), lower median positive affect (median 30, IQR 24-35 vs median 32, IQR 27-39; *P*=.01), higher median negative affect (median 16, IQR 11-24 vs median 13.5, IQR 11-18; *P*=.03), and a higher median depression score (median 8.8, IQR 4.4-13.3 vs median 5.5, IQR 2.2-12.2; *P*=.006; Table S1 in Multimedia Appendix 1).

### Sexual Health Differences by MSM Status

MSM participants had a higher mean number of female partners compared with non-MSM (mean 1.5, SD 1.7 vs mean 1.0, SD 0.6; *P*=.047) in the past 6 months. Finally, MSM participants were more likely to be diagnosed with any sexually transmitted infections (STIs) in the past 6 months (10/134, 7.5% vs 1/138, 0.7%; *P*=.005; Table S1 in Multimedia Appendix 1).

### Multivariable Analyses

Overall, the following factors were significantly associated with stimulant use: MSM status (adjusted odds ratio [aOR] 4.61, 95% CI 1.97-10.81; *P*<.001), an increasing AUDIT-C score (aOR 1.24, 95% CI 1.08-1.42; *P*=.002), increasing day-level cravings for alcohol (aOR 1.03, 95% CI 1.01-1.04; *P*<.001), depression (aOR 1.06, 95% CI 1.01-1.12; *P*=.006), number of male partners (aOR 1.32, 95% CI 1.08-1.61; *P*=.007), a greater number of female partners in the last 6 months (aOR 1.42, 95% CI 1.04-1.92), and being diagnosed with an STI (aOR 8.5, 95% CI 3.45-61.87; *P*<.001; Table S3 in Multimedia Appendix 3).

Among MSM, the following factors were significantly associated with stimulant use: an increasing AUDIT-C score (aOR 1.21, 95% CI 1.04-1.42; *P*=.01), increasing day-level cravings for alcohol (aOR 1.03, 95% CI 1.01-1.05; *P*<.001), negative affect (aOR 1.04, 95% CI 1.00-1.09; *P*=.03), depression (aOR 1.07, 95% CI 1.01-1.13; *P*=.02), number of male partners (aOR 1.31, 95% CI 1.07-1.60; *P*=.008), and being diagnosed with an STI (aOR 8.5, 95% CI 1.99-36.5; *P*=.004; Table S3 in Multimedia Appendix 3).

Among non-MSM, there were no factors significantly associated with stimulant use.

### Moderation Analyses

Results from the RERI test were significant (RERI 0.085, 95% CI 0.037-0.13; *P*<.001), providing evidence that there is a significant additive interaction between MSM status and negative affect on stimulant use. As such, the impact of negative affect on stimulant use was significantly greater among MSM compared with non-MSM.

## Discussion

### Principal Findings

Overall, our study found that compared with non-MSM, MSM had higher odds of stimulant use and the following factors associated with stimulant use: hazardous alcohol use, higher cravings for alcohol, having symptoms consistent with clinical depression, a greater number of sexual partners and being diagnosed with an STI in the past 6 months. These findings shape our recommendations for specific interventions that target these factors among MSM to reduce stimulant use burden and its consequences in this vulnerable population.

Our finding that MSM have higher odds of using stimulants is consistent with venue-based sampled studies like the National HIV Behavioral Surveillance, which observed a higher rate of stimulant use among MSM compared with the general adult population [38]. MSM are known to have higher rates of substance use overall [6-8], and our finding further confirms a need for focused attention to develop culturally competent care for MSM, especially in digital spaces [39]. The higher prevalence of stimulant use among MSM in our sample may be due to stressors associated with being a sexual minority, including but not limited to social exclusion, anti-LGBTQ+ violence, and institutionalized prejudice [40]. Future studies should assess psychosocial stressors as mediators between MSM status and stimulant use.

We also found that increasing AUDIT-C scores and day-level cravings for alcohol use were associated with stimulant use among MSM, which parallels existing literature on the relationship between hazardous alcohol consumption and stimulant use, especially among MSM [41-43]. One explanation is that the combination of alcohol and stimulants produces an intense “high” and limits the unwanted side effects that come with the use of either drug alone [44]. This finding suggests that interventions that address both stimulant use and alcohol use together may be beneficial for MSM by targeting motivators that drive the use of both substances together. For example, a promising strategy may be pharmacological interventions that reduce cravings for both substances [45]. This could help MSM reduce both alcohol and stimulant use by decreasing the need for either substance or their combined effects.

Depression was associated with stimulant use among MSM. It is known that substance use disorder and depression often occur concurrently, and individuals who experience both have worse substance use and mental health outcomes [46]. Both depression and stimulant use are more prevalent among MSM populations compared with their heterosexual counterparts [47,48]. Similarly, negative affect was associated with stimulant use among MSM in our sample. Furthermore, our moderation analysis shows that the relationship between negative affect and stimulant use is moderated by MSM status, such that the impact of negative affect on stimulant use is significantly greater among MSM compared with non-MSM. These findings linking depression and negative affect to stimulant use map onto prior research that shows a high comorbidity of mood disorders and stimulant use among MSM [49,50]. MSM may be using stimulants as a way of self-medication to combat depression, increase mood, and promote emotional stability [14,47]. Thus, interventions should focus on treating depression and promoting positive affect to reduce substance use among MSM. One example is the use of eHealth interventions, like self-monitoring via a mobile phone app, which can assess mood and substance use multiple times throughout a day and promote effective change in an individual’s natural environment [51]. By addressing the mental health motivators of stimulant use, interventions will be able to address the root causes of stimulant use more effectively and promote healthier coping mechanisms to lower the dependence on stimulant use among MSM for mental health.

A greater number of male sexual partners and being diagnosed with an STI (syphilis, gonorrhea, chlamydia, herpes simplex virus, human papillomavirus, and other) were associated with stimulant use among MSM. The use of stimulants in sexual contexts, particularly among MSM, has been documented in the literature. Stimulants are used to enhance one’s sexual experience due to their effects of increased sexual desire, energy, and confidence [52]. Anecdotally, sexual experiences while using stimulants are reported to be more pleasurable and thus a driving motivator for stimulant use among MSM [53]. Our results corroborate the consensus that stimulant use and HIV risk behaviors are inextricably linked, especially among MSM [54,55]. Reducing stimulant use among MSM is a key component of reducing HIV and other STIs. More research is needed to better understand the relationship between sexual risks and stimulant use to inform substance use and sexual health treatment and prevention efforts among MSM.

Unexpectedly, the perceived stress score was not significantly associated with stimulant use. This contrasts existing literature that has established a strong relationship between stress and substance use [56]. One possible explanation is that stressors are temporally dependent and can fluctuate quickly; it is possible that our baseline data did not accurately capture the impact of stress on substance use with a single snapshot. Future studies should measure stress more frequently in an individual’s natural environment to better capture its temporality and impact on substance use.

Additionally, lower self-esteem was not significantly associated with stimulant use. This contrasts existing literature that has found resilience (which includes high self-esteem) to be protective against substance use [57]. One possible explanation for this difference could be that most of our participants had normal to high self-esteem scores [28,29]. The small variability in self-esteem among participants may have limited our power to detect the association observed in prior studies. Additional studies including samples with a wider range of self-esteem levels may be needed to further explore its impact on stimulant use.

### Limitations

This study has several limitations. Because of our reliance on self-reported data, our study may be subject to social desirability bias. This could lead to underreporting of sensitive information like substance use and sexual behavior and threaten the validity and reliability of our results [58]. However, we mitigated this bias using standardized, computer-administered survey questions, which have been shown to increase validity in self-reported data in prior studies [59,60]. Additionally, the proportion of non-MSM who use stimulants was much smaller than MSM. Because sample size is directly correlated with statistical power, this increases the risk of committing a type II error, as our ability to detect statistically significant differences is limited by the small proportion. Our sample also lacked racial and ethnic diversity, which limits the generalizability of our findings, particularly among people of color who use stimulants. Future studies using MTurk should focus on these communities and develop ways to recruit more diverse populations to increase the applicability of research findings [61,62]. Additionally, our study’s findings are not generalizable to sexual minorities other than MSM; future studies should explore the relationship between substance use and other queer communities.

### Conclusions

In summary, this study contributes to the literature on the relationship between alcohol use, depression, negative affect, sexual health, and stimulant use among MSM in the United States. Findings from our study suggest a need to address the linkage between these factors and stimulant use to effectively lower substance use, improve mental health, and prevent HIV and other STIs among MSM in the United States. Broadly, this study also supports the feasibility of leveraging technology-based approaches to reach wider populations and increase accessibility to care.

## Appendix

Multimedia Appendix 1 Table S1. Sociodemographic, psychosocial factors, and sexual health factors at baseline among participants recruited via Amazon Mechanical Turk in the United States (N=272).

Multimedia Appendix 2 Table S2. Bivariate logistic regression models of predictors of stimulant use among Mturk recruited participants in the United States (N=272).

Multimedia Appendix 3 Table S3. Multivariable logistic regression model of predictors of stimulant use among Mturk recruited participants in the United States (N=272).

## Abbreviations

aOR: adjusted odds ratio
AUDIT-C: Alcohol Use Disorders Identification Test-Concise
CESD-10: 10-item Center for Epidemiologic Studies Depression Scale
LGBTQ+: lesbian, gay, bisexual, transgender, queer/questioning, and other
MSM: men who have sex with men
MTurk: Amazon Mechanical Turk
PANAS: Positive and Negative Affect Schedule
PSS: Perceived Stress Scale
RERI: relative excess risk due to interaction
RSES: Rosenberg Self-Esteem Scale
SAMBA: Stimulant and Alcohol Use in MTurk Behavioral Assessments
STI: sexually transmitted infection
