# Supplementary figures and images for: Stimulant Use Associated With Psychosocial Factors, HIV Risk, and Concurrent Hazardous Alcohol Use Among US Adults: Exploratory Cross-Sectional Questionnaire Study
Source: JMIR Form Res. 2023 Aug 17;7:e45717. doi: 10.2196/45717 (PMC10472175; doi:10.2196/45717)

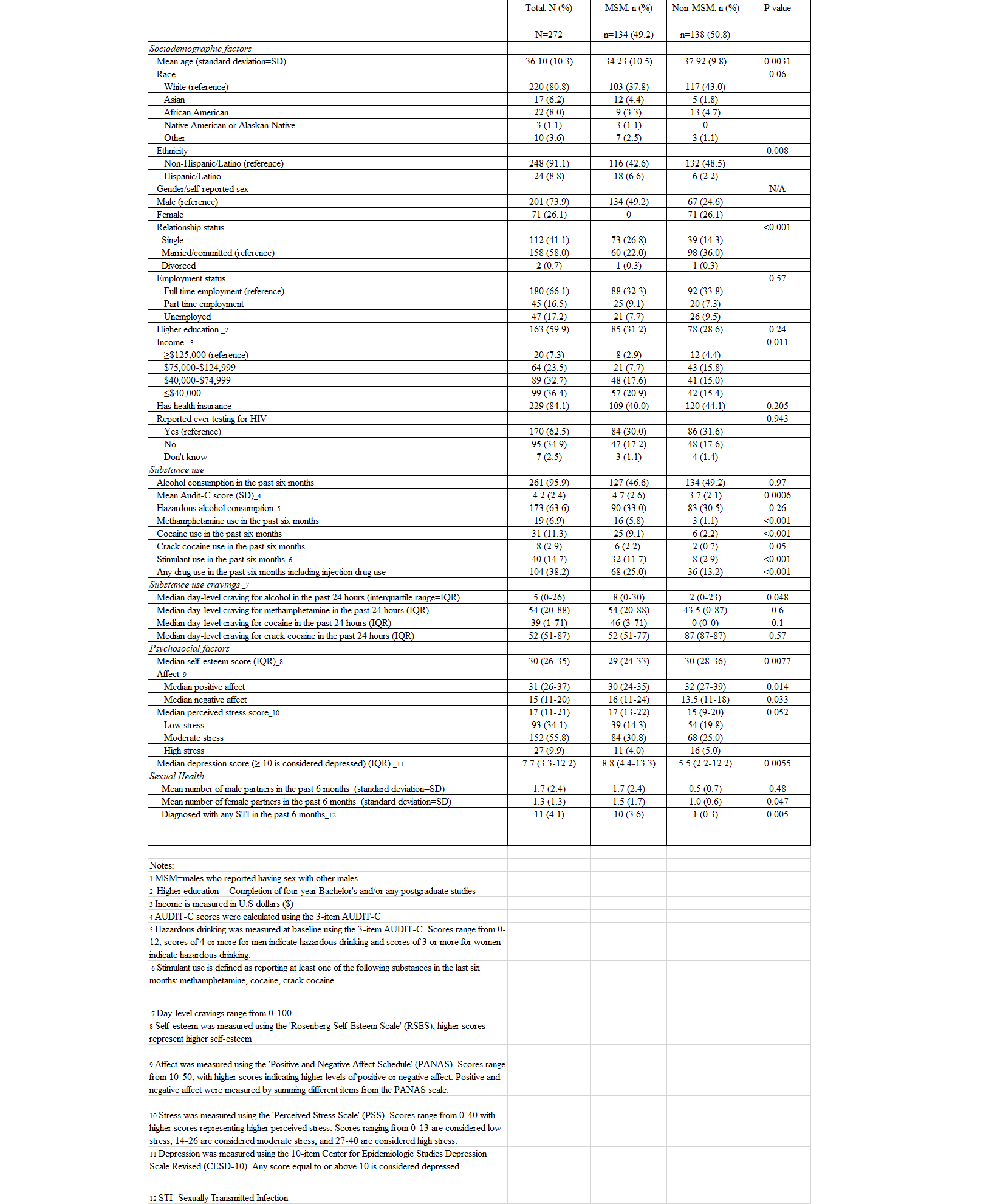

Supplement: Multimedia Appendix 1 [file formative_v7i1e45717_app1.png]

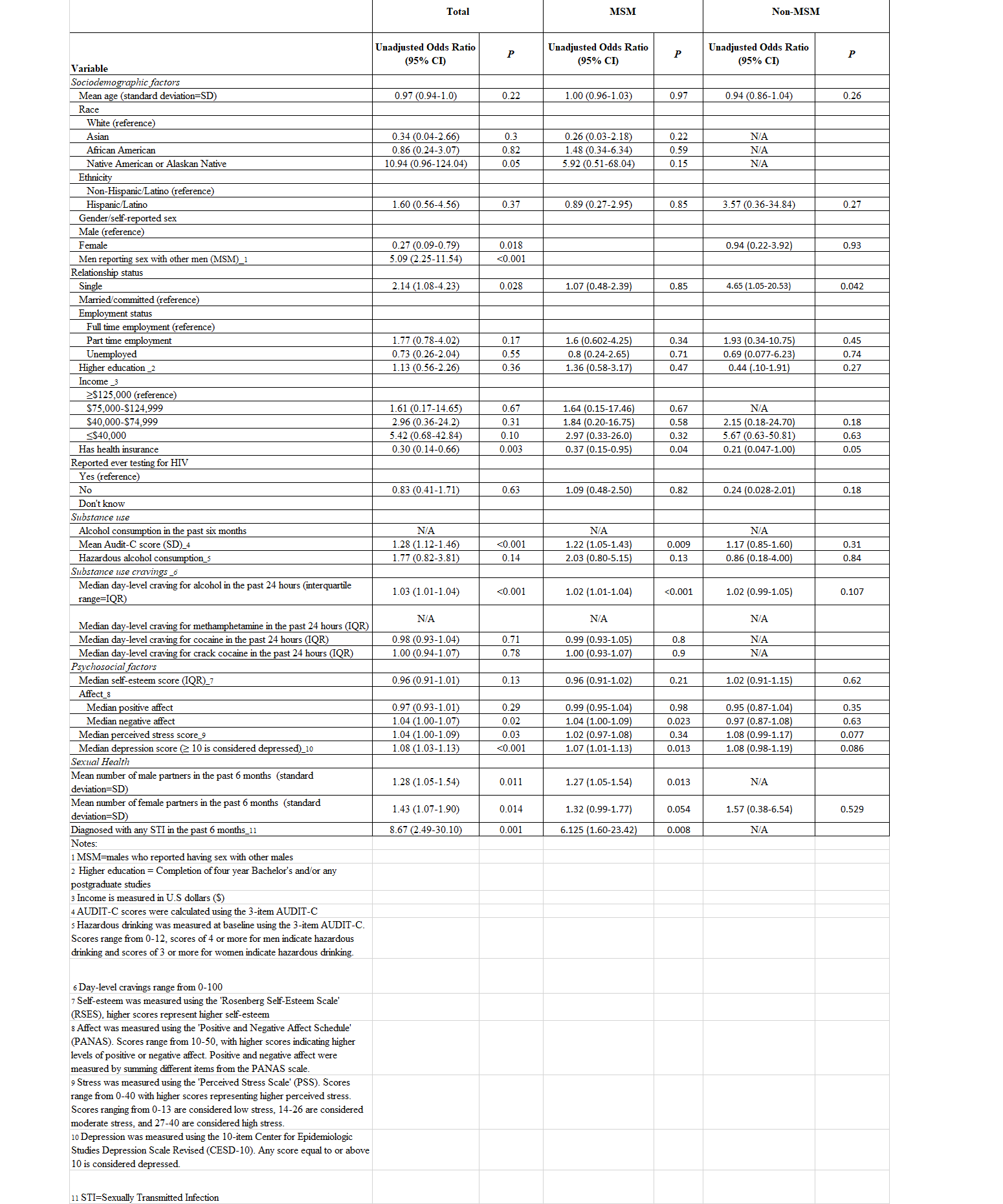

Supplement: Multimedia Appendix 2 [file formative_v7i1e45717_app2.png]

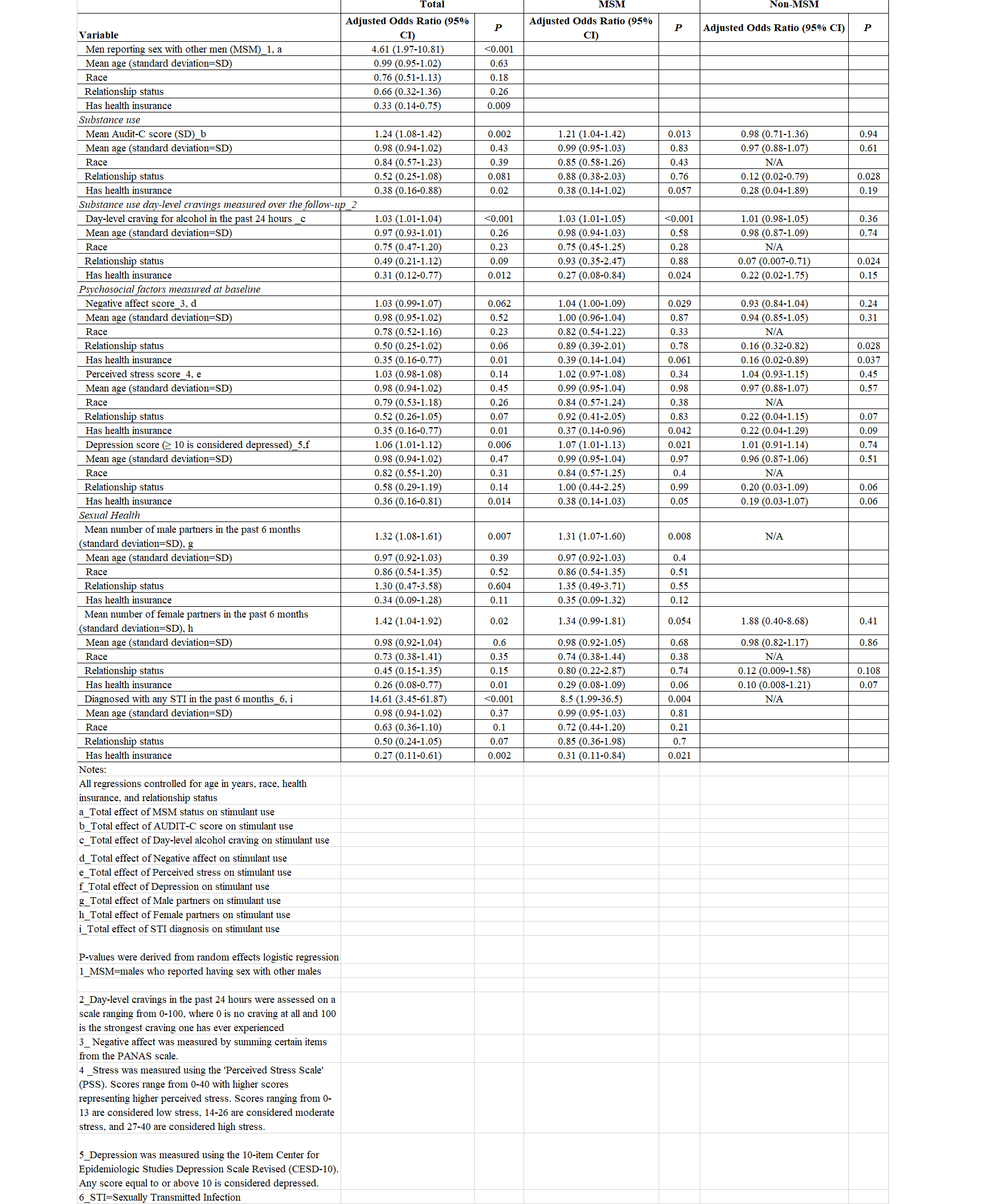

Supplement: Multimedia Appendix 3 [file formative_v7i1e45717_app3.png]
